# TC-1 Overexpression Promotes Cell Proliferation in Human Non-Small Cell Lung Cancer that Can Be Inhibited by PD173074

**DOI:** 10.1371/journal.pone.0100075

**Published:** 2014-06-18

**Authors:** Jie Lei, Wenhai Li, Ye Yang, Qiang Lu, Na Zhang, Guangzhen Bai, Daixing Zhong, Kai Su, Boya Liu, Xiaofei Li, Yunjie Wang, Xiaoping Wang

**Affiliations:** 1 Department of Thoracic Surgery, Tangdu Hospital, Fourth Military Medical University, Xi’an, Shaanxi, China; 2 Department of thoracic surgery, Shaanxi Provincial People’s Hospital, Xi’an, Shaanxi, China; Boston University Goldman School of Dental Medicine, United States of America

## Abstract

Thyroid cancer-1 (TC-1), a natively disordered protein, is widely expressed in vertebrates and overexpressed in many kinds of tumors. However, its exact role and regulation mechanism in human non-small cell lung cancer (NSCLC) are still unclear. In the present study, we found that TC-1 is highly expressed in NSCLC and that its aberrant expression is strongly associated with NSCLC cell proliferation. Exogenous TC-1 overexpression promotes cell proliferation, accelerates the cell G1-to-S-phase transition, and reduces apoptosis in NSCLC. The knockdown of TC-1, however, inhibits NSCLC cell proliferation, cycle transition, and apoptosis resistance. Furthermore, we also demonstrated that PD173074, which functions as an inhibitor of the TC-1 in NSCLC, decreases the expression of TC-1 and inhibits TC-1 overexpression mediated cell proliferation *in vitro* and *in vivo*. Nevertheless, the inhibition function of PD173074 on NSCLC cell proliferation was eliminated in cells with TC-1 knockdown. These results suggest that PD173074 plays a significant role in TC-1 overexpression mediated NSCLC cell proliferation and may be a potential intervention target for the prevention of cell proliferation in NSCLC.

## Introduction

Lung cancer is one of the leading causes of morbidity worldwide. In 2013, approximately 228,190 new cases are projected to occur in the United States, accounting for 13.74% of all new cancer cases. Even more ominously, despite the use of a multi-modality treatment, including surgical management, chemotherapy, and radiotherapy, lung cancer remains the leading cause of cancer mortality in both males and females; in fact, approximately 159,480 patients die from lung cancer-related diseases in the United States [1]. Therefore, novel therapies that arise from an improved understanding of the molecular regulators of tumor growth are urgently required. In recent years, many biomarkers that are involved in the progression of lung cancer have been investigated, but few studies have assessed the functions of TC-1 in lung cancer development.

TC-1 was originally cloned from the subtractive hybridization between a papillary thyroid carcinoma and its surrounding normal thyroid tissue [2]. It is expressed ubiquitously across a wide range of vertebrates with the highest conservation across species focused on the open reading frame (ORF). Its ubiquitous expression and sequence conservation suggest that TC-1 may play an important role in cell biology [3]. It encodes a protein of 106 amino acids without an identified functional domain, which indicates that the TC-1 protein is a member of the natively disordered protein group that has been demonstrated to perform pivotal functions in cell-cycle control, proliferation, metastasis, and signal transduction [3], [4]. An increasing number of studies have demonstrated that TC-1 is highly expressed in several carcinomas, and its aberrant expression was implicated in the proliferation of normal and cancer cells [5]–[9]. Margaret Sunde et al. confirmed that TC-1 is a novel tumorigenic protein associated with thyroid cancer and found that the overexpression of TC-1 in normal thyroid cells increased their proliferation rate, enhanced their anchorage-independent growth in soft agar, and decreased their apoptosis rate [3]. TC-1, which is located in the breast cancer-sensitive genomic region (8p^11–12^), was found to be significantly upregulated in human breast cancer cell lines and tissues, thereby implicating this protein in the development of breast cancer [8]. In gastric cancer, TC-1 was found to be one of the upregulated genes in both cell lines and carcinoma tissue, and its expression was strongly correlated with nearly all clinicopathological variables of aggressive biological behavior of cancers, including size, advanced stage, and poor survival [10], [11]. However, the expression level and the biological function of TC-1 in NSCLC have not heretofore been elucidated.

Recently, Olivier E. Pardo et al. reported that the selective fibroblast growth factor receptor (FGFR) inhibitor PD173074 blocks the proliferation and clonogenic growth of two small cell lung cancer cell lines (H69 and H510) in a dose-dependent manner and prevents FGF-2-induced chemoresistance. Surprisingly, in H510 xenografts, tumor growth was impaired with PD173074 treatment, resulting in an increase in the median survival compared with control sham-treated animals, similarly to the effect observed with the single-agent administration of cisplatin; in H69 xenografts, PD173074 induced a complete response that lasted at least 6 months in 50% of the mice. In addition, the effect of cisplatin was significantly potentiated by its coadministration with PD173074. These effects were explained by the finding that PD173074 treatment decreased intratumoral proliferation and increased cell apoptosis with a high appearance of the apoptotic cell death marker caspase-3 [12]. These encouraging findings promote further investigation of the effect of PD173074 on NSCLC cells.

To study the role of TC-1 in cell proliferation and to assess the effect of PD173074 on TC-1-mediated cell proliferation, in this study, we investigated the relationship between TC-1 expression and cell proliferation in NSCLC by immunohistochemistry, and the effect of PD173074 on TC-1-mediated cell proliferation by using a series of *in vitro* and *in vivo* loss-of-function and gain-of-function studies.

## Materials and Methods

### 2.1 Ethics Statement

The human study was approved by the Tangdu Hospital Institutional Ethics Committee, and the research study was conducted according to the provisions of the Helsinki Declaration of 2008. All of the participants provided written informed consent prior to participating in the study.

All of the animal studies were conducted with a protocol approved by the Tangdu Hospital Animal Care and Use Committee.

### 2.2 Immunohistochemistry and Evaluation

Immediately after surgical removal, samples from 122 patients with NSCLC were dissected by pathologists and snap-frozen in liquid nitrogen. The cancer samples were collected from the center of the nodules, and the normal samples were obtained from an area 5 cm distant from the nodules. Each of specimens was fixed with 4% paraformaldehyde and embedded with paraffin. The tissues were sliced to obtain 4-µm-thick sections, and the sections were dewaxed with a series of xylene and rehydrated through a graded series of alcohol. Microwave antigen retrieval was performed at 750 W for 10 min in citrate buffer (pH 6.0) to enhance the immunoreactivity. The endogenous peroxidase activity of the sections was blocked with 3% hydrogen peroxidase for 30 min, and the sections were then incubated with 5% normal goat serum in PBS for 30 min at 25°C to block any nonspecific antibody reaction. The sections were washed three times with PBS for 10 min, incubated with the primary antibodies (TC-1, 1∶100, Gene Tex, USA; Ki-67, 1∶300, Neomarkers Lab Vision Corp, CA, USA) overnight at 4°C, and then stained with an Envision™ Detection Kit (Dako, Denmark) following the manufacturer’s instructions. The sections were then treated with 0.003% 3, 30-diaminobenzidine and counterstained with hematoxylin.

The evaluation of TC-1 expression was accomplished by two pathologists without access to the clinical data and was based on both the degree of TC-1 labeling and the intensity of TC-1 staining. The degree of TC-1 labeling was measured according to the percentage of positive cells: 0 = 0–5%, 1 = 6–25%, 2 = 26–50%, 3 = 51–75%, and 4 = 76–100%. The intensity of TC-1 staining was estimated visually and stratified into four groups: 0 =  negative; 1 =  weak; 2 =  moderate; and 3 =  intense. The TC-1 score was determined as the degree of TC-1 labeling multiplied by the intensity of TC-1 staining: 0 = 0, 1+ = 1–4, 2+ = 5–8, 3+ = 9–12. Those tumors with a score of 0 were considered to be TC-1-negative, whereas the others (1+ to 3+) were considered positive. The percentages of Ki-67-reactive tumor cells were evaluated in a high-power field (400×) by counting more than 1000 tumor cells in randomly selected representative parts of the tumor [13].

### 2.3 Cell Culture

NSCLC A549, SPC-A-1, 95D, and NCI-H520 cells and the tunica mucosa bronchiorum epithelium 16HBE cells were obtained from the American Type Culture Collection (Manassas, VA, USA) and maintained in our laboratory. The cells were grown in RPMI 1640 (Gibco, Grand Island, NY, USA) supplemented with 10% fetal bovine serum (Gibco, Grand Island, NY, USA) and 100 units/mL streptomycin/penicillin and cultured at 37°C in a humidified atmosphere with 5% CO_2_. For the PD173074 experiments, A549 and A549- pLenti-shRNA1 cells were grown in serum-free and epidermal growth factor (EGF)-free medium (SITA: RPMI 1640 supplemented with 5 µg/mL insulin, 10 µg/mL transferrin, 30 nmol/L sodium selenite, and 0.25% bovine serum albumin) supplemented with PD173074 (dissolved in DMSO, Cayman, USA) at a final concentration of 1 µΜ. The growth media for the control cells were supplemented with equivalent volumes of DMSO without inhibitor.

### 2.4 Knockdown of TC-1 by RNA Interference

Four RNAi candidate target sequences to human TC-1 (Table 1) were designed and cloned into the pGCSIL-GFP vector by Shanghai GeneChem Co., Ltd. (China). TC-1 shRNA1 (Table 1) exhibited the best knockdown efficiency in 293T cells cotransfected with TC-1 and shRNA expression constructs, as revealed by western blot and immunofluorescence assays, and was thus selected for the knockdown of the endogenous TC-1 in NSCLC cells. Non-silencing-shRNA (NSRNA) was used as a negative control. The oligonucleotides encoding the TC-1 shRNA1 or NSRNA sequence and a loop sequence separating the complementary domains were synthesized and inserted into the pGCSIL-GFP by Shanghai GeneChem Co., Ltd. (China). The recombinant virus was packaged using Lentivector Expression Systems (Shanghai GeneChem Co., Ltd., China). A549 cells were infected with an enhanced infection solution and cultured in RPMI-1640 medium. One week after infection, the GFP-positive cells were sorted using a flow cytometer (Becton-Dickinson, San Jose, CA, USA). The sorted GFP^+^ cells (purity >97%) were then used in the subsequent experiments.

**Table 1:** RNAi candidate target sequences for TC-1.

Sequence name	Sequence (5′-3′)
NS-shRNA	TTCTCCGAACGTGTCACGT
TC-1 shRNA1	CTTCAGAAACTCTGGAGACAA
TC-1 shRNA 2	GACACAGCCTCTCGTAAGAAA
TC-1 shRNA 3	TGAAGAGAAGAGGATGGATAA
TC-1 shRNA 4	AGGAGAGAGCCAAGATCATTT

### 2.5 Lentivirus Construction and Transduction of Cells

The HA-TC1 construct in the pLenti-DEST vector (Invitrogen, Carlsbad, CA, USA) was described previously [14]. Briefly, an entry clone containing the full-length TC-1 was first created by our team using the pENTR directional TOPO cloning kit (Invitrogen, Carlsbad, CA, USA). After the entry clone was generated, the LR recombination reaction was performed to transfer the gene into the pLenti-DEST vector in order to create the expression clone. The construct was sequenced to ensure that the sequences and orientation were correct. The lentivirus was produced by co-transfecting 293T cells with the pLenti expression construct using the optimized packaging mix (Invitrogen, Carlsbad, CA, USA). NCI-H520 cells were transduced with lentivirus, and the pLenti-LacZ virus was used as a control. Selection was initiated 48 h after infection in RPMI-1640 medium with 10 µg/mL blasticidin in the absence of insulin or EGF. Upon reaching confluence, the selected cells were passaged and serially cultured.

### 2.6 Real Time-Polymerase Chain Reaction

The total RNA was extracted using the Trizol reagent (Invitrogen, Carlsbad, CA, USA), and the cDNA was synthesized using the PrimeScript RT Master Mix (Takara, Japan). Quantitative PCR was performed using a continuous fluorescence detecting thermal cycler ABI PRISM 7500 Sequence Detection System (ABI, CA, USA) with SYBR Premix Ex Taq II (Takara, Japan). The measurements were performed in triplicate using GAPDH as an endogenous control. qRT-PCR was performed using primers for TC-1 (5-AGCCACCAAGCCATC ATCAT-3, 5-TGTGTCGAAGTGGTAGCCATG-3) and GAPDH (5-AGGTCCACC ACTGACACGTT-3, 5-GCCTCAAGATCATCAGCA AT-3).

### 2.7 Western Blotting

Western blotting was performed as described previously [10]. Cells were harvested and washed with PBS after culture for 48 h with MG-132 (dissolved in DMSO, Cayman, America) at a final concentration of 500 nM. Equal protein amounts of the samples were separated by 10% SDS-PAGE. After blotting onto a nitrocellulose membrane (Amersham Biosciences, Piscataway, NY, USA), the membrane was incubated in blocking buffer [Tris-buffered saline (TBS), containing 0.1% Tween 20 and 5% skimmed milk] for 1 h at 25°C and then with the primary antibody (TC-1, 1∶300, Gene Tex, America; β-actin, 1∶500, Santa Cruz Biotechnology, CA, USA) at 4°C overnight. Then, the membrane was rinsed three times with TBS-T and incubated with horseradish peroxidase-labeled secondary antibodies for 2 h at 25°C. The immunoreactive bands were revealed using an enhanced chemiluminescence system (Santa Cruz Biotechnology, Santa Cruz, CA, USA), and the photographs of the bands were analyzed using FluorChemTMIS-8900 (Alpha Innotech Co., San Leandro, CA, USA).

### 2.8 MTT Assay

Cells (1×10^3^ cells/well) were seeded on 96-well plates. At a series of time points, 20 µL of MTT was added to each well, and the cells were then incubated at 37°C for 4 h. Then, 150 µL of dimethylsulfoxide (DMSO, Sigma, USA) was added to each well. The plates were shaken for 10 min, and the optical density (OD) value was measured at 490 nm using a microplate reader (Bio-Rad Model 680, USA). The cell growth curves were then drawn. All of the experiments were repeated three times, and the average values were adopted.

### 2.9 Plate Colony Formation Assay

Cells (4×10^2^ cells/well) were seeded on six-well plates and dispersed evenly by slightly shaking the plates. The cells were incubated at 37°C with 5% CO_2_ until visible colonies appeared. The colonies were fixed with 95% ethanol and stained with crystal violet staining solution. The colonies with more than 40 cells were counted using an inverted microscope, and the colony formation rate was calculated by the following formula: Plate colony formation efficiency = (number of colonies/number of cells inculcated)×100%. All of the experiments were repeated three times, and the average values were adopted.

### 2.10 Flow Cytometry Analysis of the Cell Cycle

The cells were harvested by trypsinization and collected in centrifuge tubes (2×10^6^ cells/tube). Then, 1 mL of PBS and 2 mL of dehydrated alcohol were added to each tube (4°C overnight) to fix the cells. After RNAse and PI treatment, the percentage of cells in the S phase was measured using a BD FACSAria flow cytometer (Franklin Lakes, NJ, USA). The data were analyzed using the ModFit LT software (Verity Software House, USA).

### 2.11 Flow Cytometry Analysis of Cell Apoptosis

The cells were harvested by trypsinization and collected in centrifuge tubes (1×10^6^ cells/tube). After two washes with ice-cold PBS, the cells were incubated for 15 min at room temperature in the dark in a solution containing PE-A and percp-cy5.5 (Boehringer Mannheim, Indianapolis, IN, USA) for fluorescence-activated cell sorter (FACS) analysis using a FACS instrument equipped with a doublet discriminating module (Becton-Dickinson, San Jose, CA, USA). The data were analyzed using the CellQuest software (Becton-Dickinson, San Jose, CA, USA). Ten thousand to 20,000 cells were analyzed per sample.

### 2.12 *In vivo* Tumorigenicity Assay

For the tumorigenicity assay, 4- to 6-week-old BALB/c athymic nude mice (Experimental Animal Center, The Forth Military Medical University, Xi’an, China) were subcutaneously administered 5×10^6^ A549- pLenti-shRNA1, A549- pLenti-NSRNA, NCI-H520-pLenti-TC-1, or NCI-H520-pLenti-LacZ cells. The dimensions of the tumors were measured every five days for a period of 30 days using a linear caliper. The tumor volume was calculated using the following equation: V (mm^3^) = a×b^2^/2, where “a” is the largest dimension and “b” is the perpendicular diameter. Each group included five mice. The animals were sacrificed after 30 days, and the tumors were measured and removed for further study.

### 2.13 *In vivo* PD173074 Treatment Assay

A total of 5×10^6^ A549- pLenti-shRNA1 or A549 cells (1∶1 cell suspension; Matrigel) were implanted into the flank of 4- to 6-week-old BALB/c athymic nude mice. When the tumors became measurable, 50 mg/kg PD173074/mice or an equivalent volume of buffer alone was administered daily for a total of 28 days. In addition, the mice received or did not receive two doses of 5 mg/kg cisplatin i.v. on days 1 and 15. The tumor volume was monitored using a linear caliper. The animals were sacrificed when tumor burden reached 15 mm in any dimension, and the survival was recorded using a Kaplan-Meier plot.

### 2.14 Statistical Analysis

The data are expressed as the means ± standard error (SE). The SPSS 13.0 software package (SPSS Inc, Chicago, IL, USA) was used for the statistical analyses. The Mann-Whitney U test was applied for the nonparametric data, and the Student *t* test was using for the comparisons of the means between two groups of measurement data. Analysis of variance (ANOVA) was applied for comparisons of the means of multiple groups of measurement data, and the Student-Newman-Keuls (SNK) test was used for further comparisons of each group. The log-rank (Mantel-Cox) test was used to analyze the survival curve. A P value of less than 0.05 was considered statistically significant.

## Results

### 3.1 TC-1 is Expressed at High Levels and Associated Strongly with Cell Proliferation in Human Primary NSCLC

**Figure 1:**
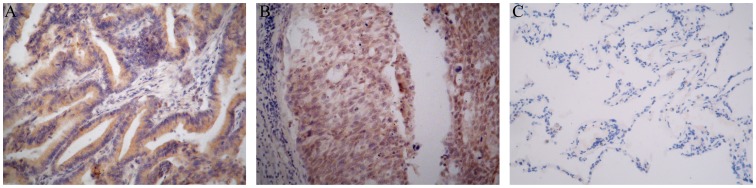
Expression of TC-1 in human NSCLC. Intensive immunohistochemical staining for TC-1 in lung adenocarcinomas (A) and squamous cell carcinomas (B). Non-reactivity for TC-1 in normal alveolar epithelial cells (C). (Magnification ×200).

To assess the expression of TC-1 in human primary NSCLC, immunohistochemistry was performed using 122 samples of NSCLC, including 68 squamous cell carcinoma and 54 adenocarcinoma samples, which were obtained from 83 males and 39 females (Table 2). TC-1 expression was evident in 49 (72.06%) of the squamous cell carcinoma samples and 41 (75.93%) of the adenocarcinoma samples. The expression of TC-1 was mostly localized in the cytoplasm, but nuclear staining was also partly present. In normal lung tissue, the non-neoplastic bronchial and alveolar epithelia cells were consistently non-reactive or low-reactive for TC-1 (Fig. 1). In addition, TC-1 expression presented slight differences between gender, age, and histological subtypes (P>0.05, Table 2).

**Table 2:** Relationship between TC-1 expression and clinicopathological characteristics in NSCLC tissues.

Clinicopathological characteristics	Score of TC-1				Z	P
	−	+	++	+++		
Gender						
Male (83)	24	27	21	11	−0.641	0.522
Female (39)	8	14	13	4		
Age (years)						
<60 (56)	14	18	16	8	−0.584	0.559
≥60 (66)	18	23	18	7		
Histological type						
SCC (68)	19	24	19	6	−1.005	0.315
AC (54)	13	17	15	9		
Ki-67 labeling index						
SCC (68)						
≤10%	17	3	2	0	−5.333	0.000
>10%	2	21	17	6		
AC (54)						
≤10%	11	2	1	1	−4.077	0.000
>10%	2	15	14	8		

The correlation between TC-1 expression and cell proliferation of NSCLC was analyzed by detecting the expression of Ki-67. As summarized in Table 2, there was a statistically significant difference between the high-proliferation group (Ki-67 labeling index >10%) and the low-proliferation group (Ki-67 labeling index ≤10%) in both the lung squamous cell carcinoma and adenocarcinoma samples (P<0.05), suggesting that TC-1 expression is strongly correlated with the cell proliferation of NSCLC.

### 3.2 Generation of Stable Clones of NSCLC Cells Overexpressing or Downregulating TC-1

**Figure 2:**
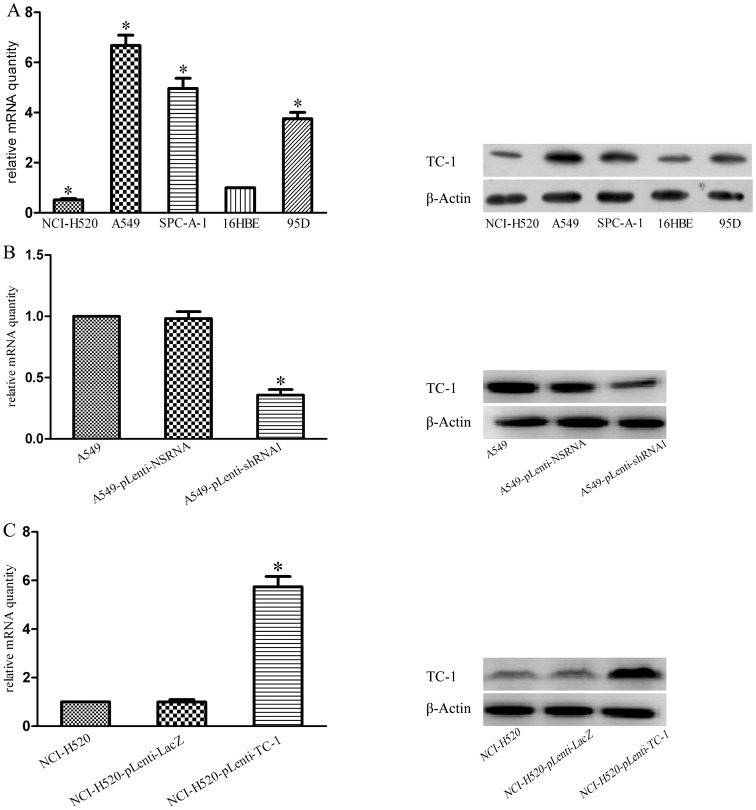
Stable clones of NSCLC cells overexpressing or downregulating TC-1 were constructed successfully. qRT-PCR and western blotting showed that A549, SPC-A-1, and 95D cells express high levels of TC-1 mRNA and protein and that NCI-H520 cells express low levels of TC-1 mRNA and protein (A). The qRT-PCR and western blotting results shown in B and C, respectively, show that TC-1 mRNA and protein are significantly downregulated in A549-pLenti-shRNA1 cells and significantly upregulated in NCI-H520-pLenti-TC-1 cells compared with the controls. The columns represent the average of the relative mRNA quantity from at least three independent experiments. The bars show the SE. *indicates statistically significant changes (P<0.05) among five groups (A) or three groups (B, C).

To study the effect of TC-1 aberrant expression on NSCLC cells, qReal-Time PCR and western blotting were performed using the human NSCLC cell lines A549, SPC-A-1, 95D, and NCI-H520, and the 16HBE cell line was used as a control group. As shown in Fig. 2A, the expression level of TC-1 in A549, SPC-A-1, and 95D cells was higher than that obtained in the 16HBE cells, and the expression level of TC-1 in NCI-H520 cells was lower than that observed in the 16HBE cells. Based on these results, A549 and NCI-H520 were found to be representative cells that exhibit the highest and a lower expression level of TC-1 and were selected for further study. Then, pLenti-shRNA1 was transfected into A549 cells, and pLenti-TC-1 was transfected into NCI-H520 cells. Stable clones were isolated after clone screening by fluorescence-activated cell sorting or blasticidin, respectively. The qReal-Time PCR and western blotting results showed that TC-1 expression was markedly decreased or increased in the treated cells compared with the control, whereas the negative control did not exhibit any change in the level of TC-1 expression (Figs. 2B and 2C).

### 3.3 TC-1 Promotes Cell Proliferation and Cell Cycle Transition and Inhibits Cell Apoptosis in NSCLC *in vitro*

**Figure 3:**
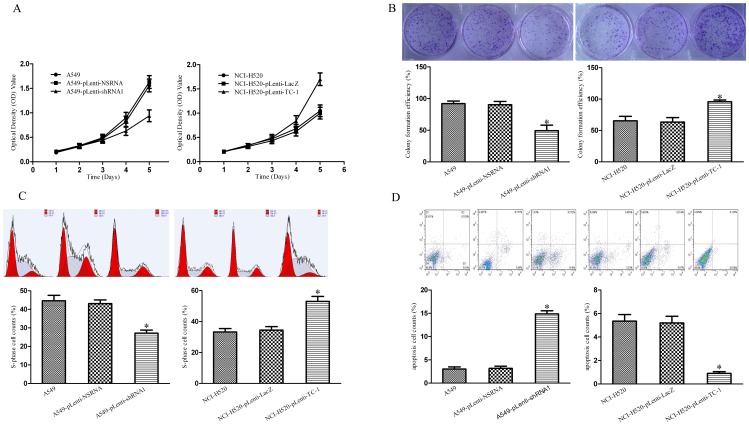
TC-1 promotes NSCLC cell proliferation, cell cycle transition, and apoptosis resistance *in vitro*. (A) MTT assay. The optical density value was detected at a series of time points to evaluate cell proliferation. (B) Plate colony formation assay. The colonies were stained with crystal violet staining solution and counted, and the clone formation rate was then calculated. (C) Cell cycle assay. The percentage of cells in the S phase was measured using a flow cytometer, and the data were analyzed using the ModFit LT software. (D) Cell apoptosis assay. The cells were incubated in the dark in a solution containing PE-A and PerCP-Cy5.5. The percentage of apoptotic cells (lower right quadrant) was analyzed using a FACS equipped with a doublet discriminating module, and the data were analyzed using the CellQuest software. The columns represent the average colony formation rate (B), S-phase cell rate (C), and apoptosis cell rate (D) from at least three independent experiments. The bars show the SE. *indicates statistically significant changes (P<0.05) among three groups.

To investigate the function of TC-1 expression in NSCLC cell proliferation, loss-of function and gain-of function studies were performed. Notably, in the MTT assay, the transfection of pLenti-TC1 transfection enhanced the proliferation of NCI-H520 cells compared with the control cells. Alternatively, TC1 knockdown using TC1-shRNA1 showed significant downregulation of proliferation in A549-pLenti-shRNA1 cells compared with NSRNA-transfected cells (Fig. 3A). Furthermore, the colony numbers of the pLenti-TC-1 and pLenti-NS RNA groups were higher than those obtained for the pLenti-LacZ and pLenti-shRNA1 groups, respectively (Fig. 3B). These results suggest that TC-1 promotes cell proliferation in NSCLC *in vitro*.

The cell cycle phase distribution was measured by flow cytometry. The results showed that the percentage of cells in the S phase was significantly increased after treatment with pLenti-TC1 compared with that obtained after treatment with pLenti-LacZ. In contrast, the percentage of cells in the S phase decreased significantly after treatment with pLenti- shRNA1 compared with that obtained with pLenti-NSRNA (Fig. 3C). These results indicate that TC-1 promotes the G1-to-S-phase transition.

Moreover, the effect of TC-1 on NSCLC cell apoptosis was analyzed by flow cytometry. As shown in Fig. 3D, the cells transfected with pLenti-TC-1 displayed lower apoptotic rates compared with the cells transfected with pLenti-LacZ, and the cells transfected with pLenti-shRNA1 displayed higher apoptotic rates compared with the cells transfected with pLenti-NSRNA, suggesting that TC-1 inhibits cell apoptosis in NSCLC.

### 3.4 TC-1 Promotes NSCLC Cell Proliferation *in vivo*

**Figure 4:**
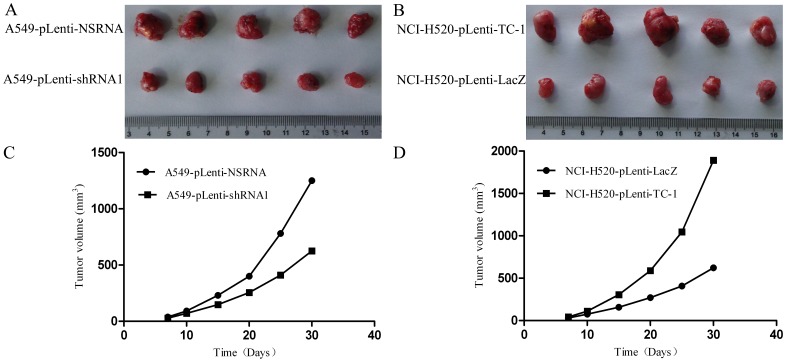
TC-1 has a significant impact on NSCLC cell proliferation *in vivo*. Subcutaneous tumor model (n = 5). Cells were subcutaneously injected into the flank of athymic nude mice, and the tumor volume was recorded every five days (C, D). The mice were sacrificed 30 days after injection, the tumors were removed, and photographs were taken (A, B).

To assess the function of TC-1 expression in NSCLC cell proliferation *in vivo*, an *in vivo* tumorigenicity assay was performed. The dimensions of the tumors were measured every five days for a period of 30 days (Figs. 4B and 4D). At the end of the experiment, the mice were sacrificed, and the tumors were removed and photographed (Figs. 4A and 4C). As shown in Fig. 4, our tumorigenicity assay results indicate that the upregulation of the expression level of TC-1 promotes cell proliferation *in vivo*, whereas the knockdown of the expression level of TC-1 inhibits cell proliferation *in vivo*.

Part of each subcutaneous nodule was sectioned and subjected to immunohistochemistry for Ki-67. Our results showed that there was a significant difference in the number of Ki-67-reactive tumor cells in the subcutaneous nodules between the two groups (the Ki-67 indexes of the subcutaneous nodules were the following: A549- pLenti-NSRNA, 68.98±2.56%; A549- pLenti-shRNA1, 28.63±2.38%; P<0.05; NCI-H520- pLenti-TC-1, 86.26±3.16%; NCI-H520- pLenti-LacZ, 51.34±1.78%; P<0.05). These findings indicate that TC-1 significantly enhances the proliferation capacity of NSCLC cells *in vivo*.

### 3.5 PD173074 Decreases the Expression of TC-1 in a Dose-depending Manner Over a Certain Range

**Figure 5:**
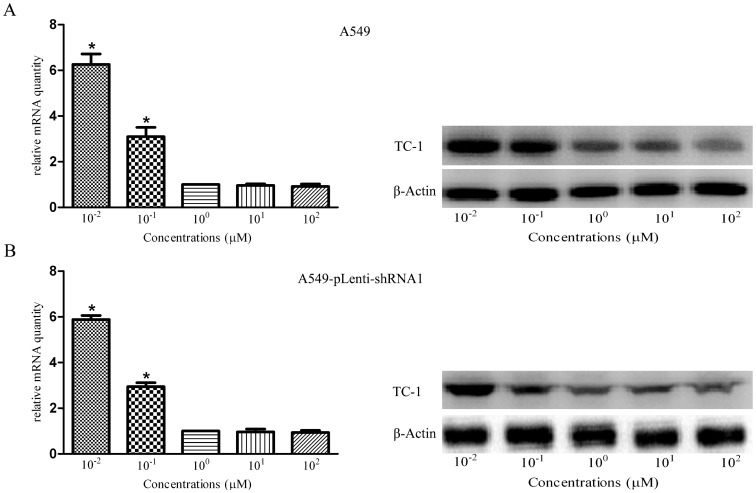
PD173074 inhibits the expression of TC-1 in A549 and A549-pLenti-shRNA1 cells. The qRT-PCR and western blotting results showed that the expression level of TC-1 decreased with an increase in the concentration of PD173074 and levels out when the concentration of PD173074 reaches 1 µΜ in A549 (A) and A549-pLenti-shRNA1 (B) cells. The columns represent the average of the relative mRNA quantity from at least three independent experiments. The bars show the SE. *indicates statistically significant changes (P<0.05) among five groups.

To test whether the addition of PD173074 influences the expression of TC-1 in NSCLC, A549 and A549-pLenti-shRNA1 cells in SITA were treated with PD173074. The results of RT-PCR and western blotting showed that the expression level of TC-1 decreased with an increase in the concentration of PD173074 and levels out when the concentration of PD173074 reaches 1 µΜ (Fig. 5). The concentration of 1 µΜ was thus selected for further study.

### 3.6 PD173074 Inhibitions of Cell Proliferation, Cycle Transition, and Apoptosis Resistance Depends on the TC-1 Expression Level *in vitro*

**Figure 6:**
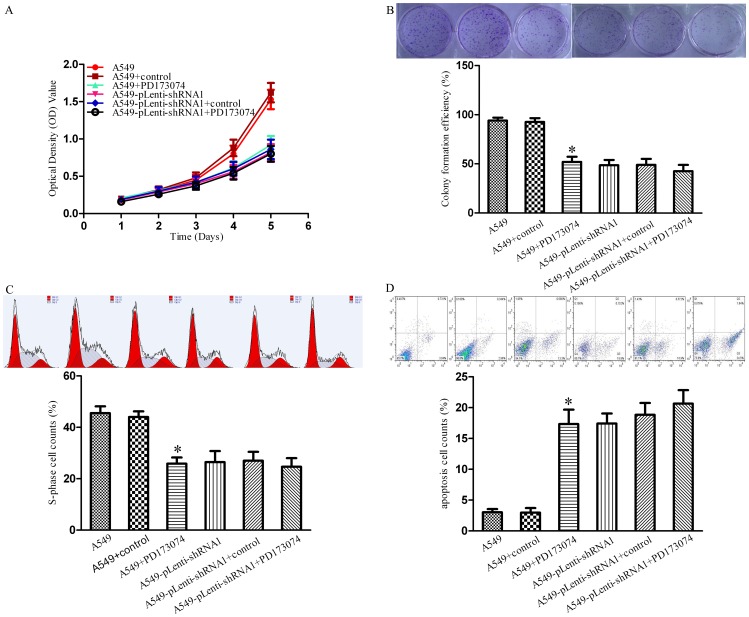
PD173074 inhibition of cell proliferation, cycle transition, and apoptosis resistance depends on the TC-1 expression level *in vitro*. (A) MTT assay. The optical density value was detected at a series of time points to evaluate cell proliferation. (B) Plate colony formation assay. The colonies were stained with crystal violet staining solution and counted, and the clone formation rate was then calculated. (C) Cell cycle assay. The percentage of cells in the S phase was measured using a flow cytometer, and the data were analyzed using the ModFit LT software. (D) Cell apoptosis assay. The cells were incubated in the dark in a solution containing PE-A and PerCP-Cy5.5. The percentage of apoptotic cells (lower right quadrant) were analyzed using a FACS equipped with a doublet discriminating module, and the data were analyzed using the CellQuest software. The columns represent the average colony formation rate (B), S-phase cell rate (C), and apoptosis cell rate (D) from at least three independent experiments. The bars show the SE. *indicates statistically significant changes (P<0.05) between the A549 group and the A549+PD173074 group.

To study the effect of PD173074 on TC-1-mediated cell proliferation in NSCLC, an MTT assay, plate colony formation assay, cell cycle analysis, and cell apoptosis analysis were performed. As shown in Fig. 6A, PD173074 treatment has a marked influence on the cell growth curves of the untransfected cells, as illustrated by the difference between the A549+PD173074 group and the A549 group, but has little influence on the cell growth curves of the transfected cells, as indicated by the difference between the A549-pLenti-shRNA1+PD173074 group and the A549-pLenti-shRNA1 group. A similar result was observed in the plate colony formation assay: there was a significant difference in the colony formation rate between the A549+PD173074 group and the A549 group, but only slight differences in the colony formation rate were observed between the A549-pLenti-shRNA1+PD173074 group and the A549-pLenti-shRNA1 group (Fig. 6B). As shown in Fig. 6C, the percentages of cells in the S phase in the populations of PD173074-treated A549 cells, A549 cells, PD173074-treated A549-pLenti-shRNA1 cells, and A549-pLenti-shRNA1 cells were 29.47±0.62%, 49.5±1.89%, 28.02±0.97%, and 29.5±1.02%, respectively. Obviously, there was a significant difference between the A549+PD173074 group and the A549 group, but only slight difference between the A549-pLenti-shRNA1+PD173074 group and the A549-pLenti-shRNA1 group. As shown in Fig. 6D, the apoptotic rates of the PD173074-treated A549 cells were significantly higher than that of the A549 cells; however, there was no marked difference in the apoptotic rate between the PD173074-treated A549-pLenti-shRNA1 cells and A549-pLenti-shRNA1 cells. Taken together, these data indicate that PD173074 inhibits TC-1-mediated cell proliferation, cell cycle transition, and cell apoptosis resistance when TC-1 is highly or moderately expressed but not when it is lowly expressed.

### 3.7 PD173074 Inhibitions of Cell Proliferation, Cycle Transition, and Apoptosis Resistance Depend on the TC-1 Expression Level *in vivo*

**Figure 7:**
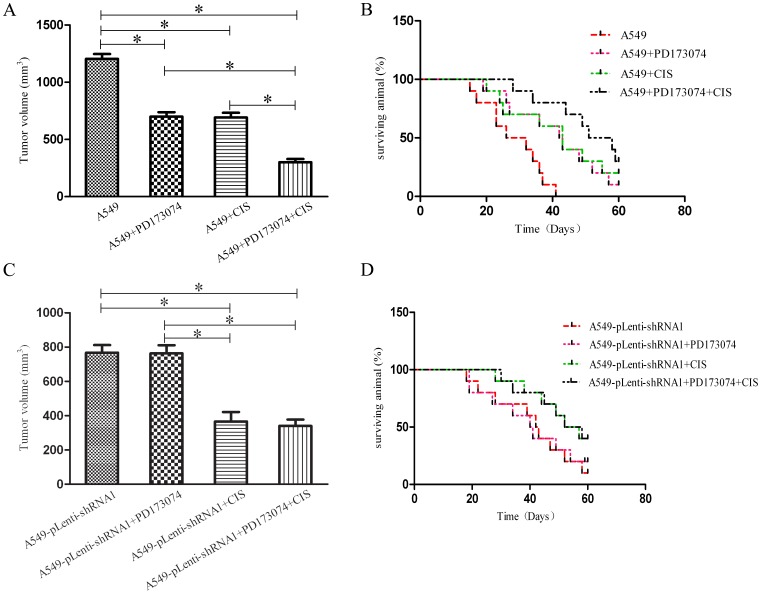
PD173074 inhibition of cell proliferation, cycle transition, and apoptosis resistance depends on the TC-1 expression level *in vivo*. Subcutaneous tumor model (n = 10). A549- pLenti-shRNA1 or A549 cells were implanted into the flank of nude mice. PD173074 or buffer alone was administered daily for 28 days. In addition, the mice received or did not receive two doses of 5 mg/kg cisplatin i.v. on days 1 and 15. (A, C) The tumor volumes were scored 28 days after the first administration of PD173074. (B, D) The animal survival was recorded as a Kaplan-Meier plot. The columns represent the average tumor volume. The bars show the SE. *indicates statistically significant changes (Student’s t test, P<0.05) between two groups. CIS, Cisplatin.

To further survey the effect of PD173074 on TC-1-induced cell proliferation in NSCLC, a series of experiments were performed *in vivo*. Cisplatin, the basic drug used for the chemotherapy of NSCLC, was used as the control. As shown in Figs. 7A and 7B, compared with the MOCK group, the PD(+), CIS(+), and PD+CIS(+) groups exhibited significant inhibition of A549 cell proliferation and prolonged survival of the tumor-bearing animals, particularly the PD+CIS(+) group, which presented an additional inhibitory effect and survival benefit compared with the other groups. In fact, PD173074 alone was as efficient as cisplatin in reducing the tumor growth rate and prolonging the survival of tumor-bearing animal compared with the Mock group. However, as shown in Figs. 7C and 7D, compared with the MOCK group, only CIS(+) and PD+ CIS(+) groups significantly inhibited A549-pLenti-shRNA1 cell proliferation and prolonged the survival of tumor-bearing animals, and the PD(+) group failed to inhibit A549-pLenti-shRNA1 cell proliferation and to prolong the survival of tumor-bearing animals with statistical significance. These results also indicate that PD173074 plays a role in the regulation of TC-1-induced proliferation when TC-1 is highly or moderately expressed. In this case, the effects caused by PD173074 alone were equivalent to the effects of cisplatin, and the combination of PD173074 with cisplatin may improve the chemotherapy effectiveness of cisplatin in NSCLC.

## Discussion

A growing body of evidence shows that TC-1 is an oncogene that is overexpressed in several types of neoplasm [2]–[4], [7]–[11]. In some cancers, TC-1 expression has been proven to be a valuable marker for malignance clinicopathological characteristics [3], [10]–[11], [14]. In this study, the immunohistochemical results showed that TC-1 expression was evident in 90 (73.77%) of the NSCLC tissues and in 18 (14.75%) of the normal lung tissues. There was a marked upregulation of TC-1 expression in NSCLC tissues compared with normal lung tissues. Moreover, further analysis found that the expression level of TC-1 in the Ki>10% group was higher than that in the Ki≤10% group in both the squamous cell carcinoma and adenocarcinoma samples. Consistent with thyroid cancer, gastric cancer, and breast cancer, our data revealed that TC-1 is highly expressed in NSCLC tissues, and its overexpression was strongly correlated with cell proliferation. Similar results were also obtained with NSCLC cell lines. We examined four NSCLC cell lines for TC-1 expression by real-time RT-PCR and western blotting, results showed that three of the cell lines, particularly A549 cells, exhibit a higher expression of TC-1 compared with the control (16HBE cells) and that only one cell line (NCI-H520) presents a lower expression of TC-1. A series of loss-of function and gain-of-function studies both *in vitro* and *in vivo* confirmed that the forced overexpression of TC-1 enhances cell proliferation, accelerates the cell G1-to-S-phase transition, and reduces cell apoptosis, whereas the downregulation of the expression of TC-1 gave the reverse results. Thus, we affirmed that TC-1 is an oncogene in NSCLC that promotes cell proliferation, cell cycle transition, and apoptosis resistance and may be a valuable marker for the occurrence and development of NSCLC.

An important finding of our study is that PD173074 inhibits TC-1 overexpression mediated cell proliferation in NSCLC. PD173074, a small-molecular-weight pharmacological tyrosine kinase inhibitor [15], has reportedly shown powerful inhibitory effects on cell proliferation [12], [14], [16]–[21]. In breast cancer, PD173074 treatment markedly reduces proliferation and promotes apoptosis in MKN45 cells. Moreover, the effects of the combination of PD173074 and 5-fluorouracil in inhibiting proliferation, increasing apoptosis were superior to these effects following the single agent treatments [16]. Sara A. Byron et al. showed that treatment with PD173074 results in cell cycle arrest and induction of cell death in endometrial cancer cells with activating mutations in FGFR2 [17]. The similar results were found in breast cancer [14], [18]. In bladder cancer, PD173074 markedly suppresses cell proliferation in two cell lines (UM-UC-14 and MGHU3) that express the mutated FGFR3 protein *in vitro* and *in vivo*, and further analysis revealed that the growth inhibitory effect of PD173074 is associated with arrest at G_1_-S transition and induction apoptosis in a dose-dependent manner [19]. PD173074 inhibits the MAPK pathway, which regulates the activity of AP-1 and induces the mesenchymal-epithelial transition (MET), and this induction of MET likely suppresses head and neck squamous cell carcinoma cell proliferation and invasion [20]. Lamont FR et al found that the small molecule FGF receptor inhibitor PD173074 block FGFR-dependent urothelial carcinoma growth *in vitro* and *in vivo* [21]. Moreover, in SCLC, PD173074 inhibits H-510 and H-69 cell proliferation and prevents FGF-2-induced chemoresistance [12]. In the current study, we treated A549 and A549-pLenti-shRNA1 cells with different concentrations of PD173074, qRT-PCR and western blotting showed that the expression level of TC-1 decreased significantly until the concentration of PD173074 reached 1 µΜ, which suggests that PD173074 inhibits the expression of TC-1 in a dose-dependent fashion over a certain range. This finding is supported by a previous study, which determined the gene expression profile in SUM 52 cells after exposure to PD173074 using a 20,000-element cDNA array. Examination of the expression profile revealed that the TC-1 mRNA levels were dramatically reduced, and this result was corroborated by northern blot hybridization and Q-RT-PCR [14]. Furthermore, we found that PD173074 dramatically blocks cell proliferation, suppresses cell cycle transition, and promotes cell apoptosis in A549 cells but not in A549- pLenti-shRNA1 cells *in vitro*; consistently, PD173074 treatment exerted a marked inhibitory effect against the proliferation of A549 cells but a slight effect in A549- pLenti-shRNA1 cells *in vivo*, which reveals that the inhibitory effect of PD173074 in NSCLC is related to the TC-1 expression levels. Thus, cell lines with TC-1 knockdown were unresponsive to PD173074, whereas cell lines that overexpression of TC-1 were highly sensitive to this inhibitor in NSCLC. In addition, there was an encouraging *in vivo* result: PD173074 as a single agent was as efficient as cisplatin at inhibiting cell proliferation and prolonging the survival of A549 tumor-bearing nude mice, and the combination of the inhibitor with cisplatin exerted an improved effect. This result was consistent with the previous research and suggests that the inhibition of TC-1-induced proliferation by PD173074 may provide a powerful therapeutic strategy for NSCLC treatment in a clinical setting (at least by improve the chemotherapeutic effect of cisplatin in NSCLC patient with TC-1 overexpression). Of course, further studies need to be performed in the future to confirm our results, including the exact mechanisms of TC-1, PD173074 and the relationships between TC-1 and the FGFR family, which has been demonstrated to be susceptible to the inhibitor [14], [16]–[23].

In conclusion, our study revealed that TC-1 is a newly identified oncogene in NSCLC. Overexpression of TC-1 occurs in 73.77% of the primary lung cancer tumor specimens and correlates strongly with NSCLC cell proliferation. Furthermore, the role of TC-1 overexpression in cell proliferation, cell cycle transition, and apoptosis resistance can be blocked by PD173074. The inhibition effect of PD173074 is mechanistically linked to the expression level of TC-1. Further studying the precise mechanism of PD173074 treatment and TC-1 regulation network will inspire us to find new ways to prevent tumor growth.
